# A cell-based assay for rapid assessment of ACE2 catalytic function

**DOI:** 10.1038/s41598-023-41389-7

**Published:** 2023-08-29

**Authors:** Warren M. Meyers, Ryan J. Hong, Wun Chey Sin, Christine S. Kim, Kurt Haas

**Affiliations:** 1 Djavad Mowafaghian Centre for Brain Health, University of British Columbia, Vancouver, BC Canada; 2 Department of Cellular & Physiological Sciences, University of British Columbia, Vancouver, BC Canada; 3 School of Biomedical Engineering, University of British Columbia, Vancouver, BC Canada

**Keywords:** Biochemical assays, High-throughput screening, Biochemistry, Biological techniques, Pharmacogenetics

## Abstract

Angiotensin-converting enzyme II (ACE2) is a monocarboxypeptidase expressed throughout multiple tissues and its catalysis of bioactive peptides regulates the renin-angiotensin system mediating blood pressure homeostasis. ACE2 is implicated in a variety of diseases, including obesity, diabetes, and cardiovascular diseases, and is the obligate entry receptor for SARS-CoV-2 infection. Disease-associated genetic variants of ACE2 are increasingly being identified but are poorly characterized. To aid this problem, we introduce a fluorometric cell-based assay for evaluating surface-expressed ACE2 catalytic activity that preserves the native glycosylation of the host environment and is amenable to high-throughput analysis of ACE2 variants in multi-well plates. We demonstrate sensitivity to detecting catalysis of the key ACE2 substrates, Angiotensin II, Apelin-13, and des-Arg^9^-bradykinin, and impact of a catalytically-deficient ACE2 variant. Normalizing catalytic measures to surface ACE2 expression accounts for variability in ACE2 variant transfection, surface delivery or stability. This assay provides a convenient and powerful approach for investigating the catalytic characteristics of ACE2 variants involved in cardiovascular peptide cascades and homeostasis of multiple organs.

## Introduction

Angiotensin converting enzyme 2 (ACE2) is an integral type I transmembrane protein with monocarboxypeptidase activity that is expressed on the cell surface of a wide diversity of cell types and tissues including the lung, liver, small intestine, adipose tissue, kidney, heart, and to a limited extent in the brain^1,2^. ACE2 is best recognized as a critical regulator of the renin-angiotensin system (RAS) where it regulates blood pressure homeostasis by converting the vasoconstrictor Angiotensin II (Ang II) into the vasodilator Angiotensin (1–7) (Ang1-7) by removing its terminal phenylalanine^1,3,4^. Functionally, ACE2 opposes the actions of angiotensin converting enzyme (ACE) which converts Angiotensin I to Angiotensin II leading to vasoconstrictive, inflammatory and fibrotic effects via the AT1 receptor^1,3^. ACE2-mediated increase in Ang1-7 promotes enhanced signaling via the AT1-7/Mas receptor which has systemic vasodilatory, anti-inflammatory and anti-fibrotic effects on lung epithelium and other cells throughout the body^5–7^. In addition to these well-characterized effects on Ang II, ACE2 processes and deactivates the bioactive peptides Apelin and des-Arg9-bradykinin (des-Arg^9^-BK) independent of the RAS pathway^8^. Apelin functions primarily as a systemic vasodilator resulting in lowered blood pressure but has been shown to cause vasoconstriction in some tissue types^9,10^ whereas des-Arg^9^-BK signals through the bradykinin-1 receptor promoting pro-inflammatory and vasodilatory effects^11^. ACE2 has been linked to multiple disease states, including obesity^12^, diabetes^13,14^, and cardiovascular diseases^15–17^, COVID-19 infection^18,19^, comorbidities associated with poor COVID-19 outcomes^20–23^, as well as long-haul COVID-19 pathophysiology^4^. Given the large number of polymorphisms of ACE2 in the general population^24–26^ and recent implication of ACE2 single nucleotide variants and disease^14,27–29^, high-throughput biological methods and assays are needed to examine the relationship between ACE2 genotype and function, and their potential links to disease susceptibility and expression.

ACE2 monocarboxypeptidase activity can be assessed using fluorometric-based measures of the cleavage of phenylalanine on Ang II and Ap-13 substrates, which has been used to assess catalytic activity of recombinant, soluble ACE2^30^. Building on these methods, we present an assay for ACE2 catalytic function based on cell-surface ACE2 in cultured cells in a transient transfection context. Our approach preserves human-specific glycosylation of the native host environment and is amenable for high-throughput assessment of large ACE2 variant libraries. We demonstrate the assay’s sensitivity to catalysis of the substrates Angiotensin II (Ang II), Apelin-13 (Ap-13), and des-Arg^9^-BK, inhibition by specific ACE2 inhibitors, and for assessment of an ACE2 variant lacking catalytic activity. An important feature of this assay is a 3xHA tag marker for ACE2 surface expression that is used to normalize catalysis measurements, allowing the assessment of ACE2 variant libraries via acute transfection, and to accommodate ACE2 variants with altered surface delivery or stability.

## Results

**Figure 1:**
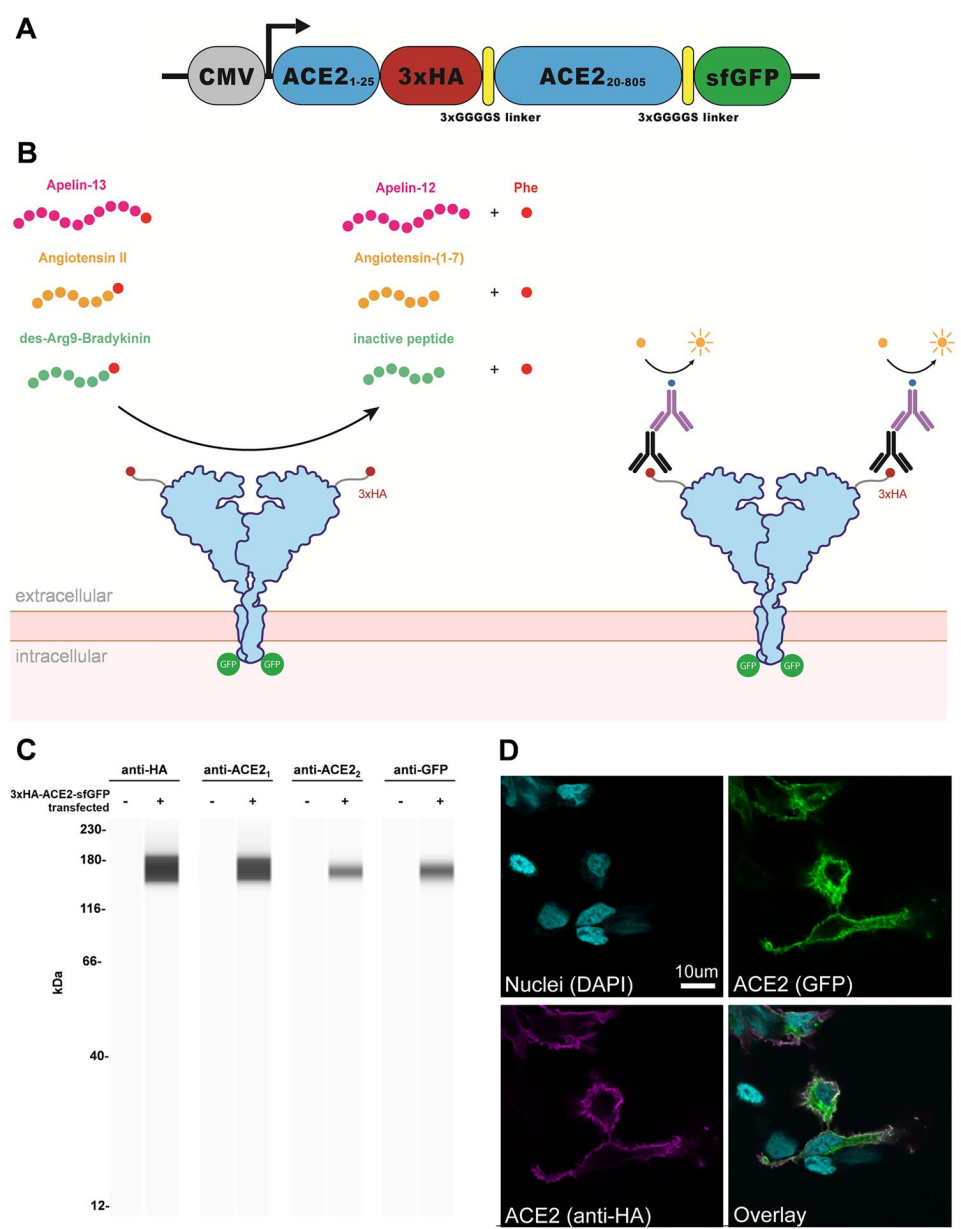
Design for a membrane-localized ACE2 expression system. (**A**) Our ACE2 construct is driven by a CMV promoter followed by the first 25 residues of ACE2 containing the leader sequence that direct ACE2 to the plasma membrane. This is followed by a 3xHA tag linked to the remainder of ACE2 (20–805) and a C-terminal sfGFP. Both 3xHA and sfGFP fusions are separated from ACE2 by flexible 3xGGGGS linkers. (**B**) The ACE2 fusion protein is designed to be embedded in the plasma membrane where it can perform extracellular carboxypeptidase-mediated metabolism and its levels can be detected by cell staining with antibodies to HA. (**C**) Lysates from untransfected or 3xHA-ACE2-sfGFP-transfected HEK293 cells were analyzed by automated Jess capillary immunoassay using antibodies to HA, GFP, and two ACE2 antibodies. (**D**) Confocal fluorescence microscopy of HEK cells transfected with 3xHA-ACE2-sfGFP and stained with HA and the nuclear stain DAPI.

To develop a cell-based assay for ACE2 activity we inserted an internal 3xHA epitope tag following the N-terminal signal peptide region (AA 1–25) that is cleaved during receptor processing and membrane insertion (Fig. 1A). This design allows for native membrane trafficking and expression of an extracellular 3xHA tag which is used to normalize for surface expression (Fig. 1B). A super-folder GFP (sfGFP) fusion was also added to the C-terminus as a marker for total ACE2 expression. This dual-tagged *ACE2* cassette was placed in an expression plasmid under the control of a CMV promoter (CMV-3xHA-ACE2-sfGFP).

To assess exogenous expression of our ACE2 construct, we transfected ACE2 plasmid constructs in human embryonic kidney (HEK) cells. Cell lysates were probed with antibodies against HA, GFP, and two different epitopes of ACE2 using an automated capillary immunoassay (Jess, Protein Simple) (Fig. 1C). Electrophoretic analysis of the lysates revealed a single species with an estimated molecular weight of ~ 160 kDa only in transfected cells, indicating absence of detectable endogenous ACE2 in HEK cells. Using confocal microscopy, we further demonstrated that HA co-localized with GFP at the plasma membrane when stained under non-permeabilized conditions (Fig. 1D).

**Figure 2:**
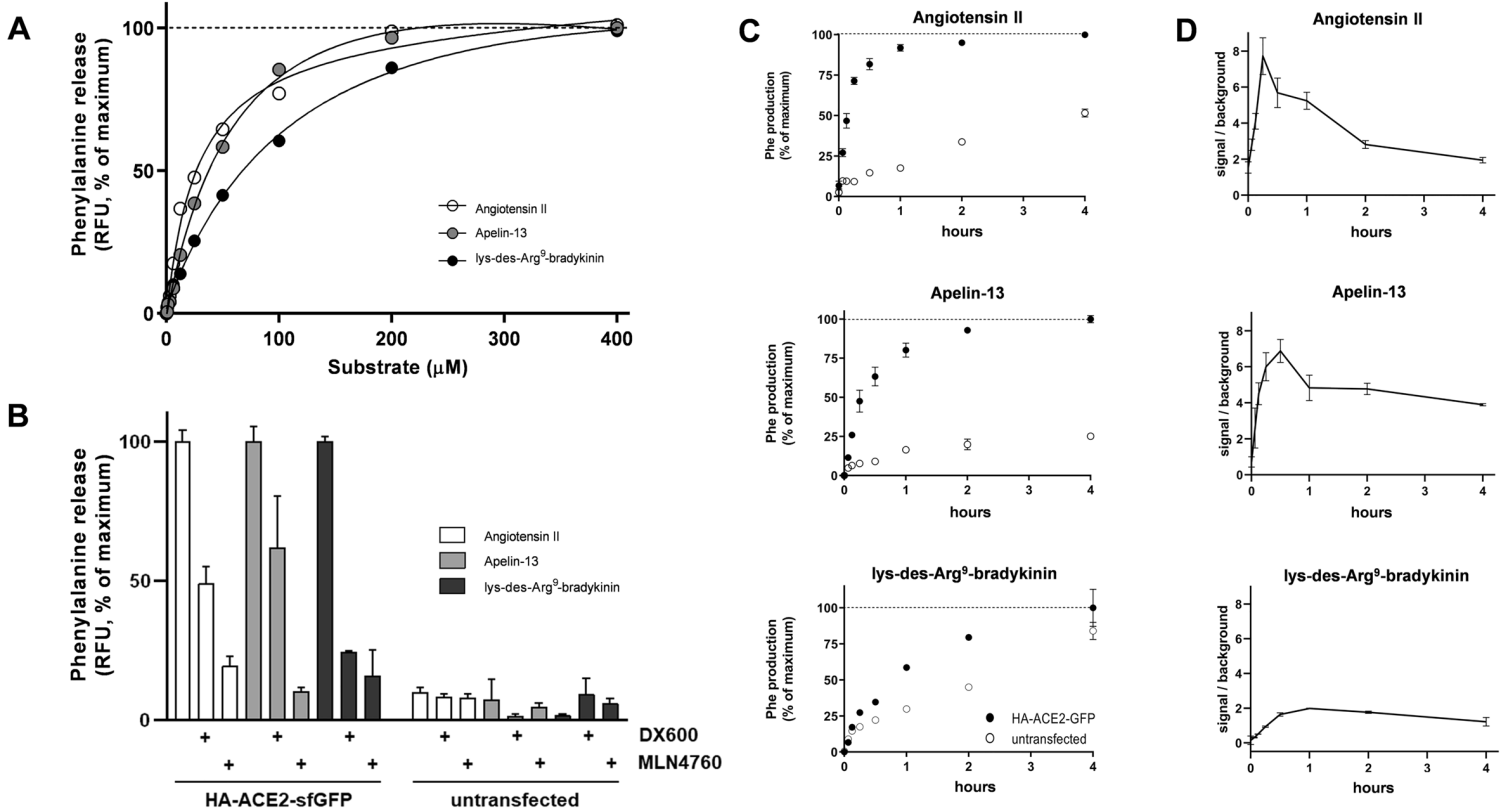
Sensitivity, specificity and time course of exogenous ACE2 carboxypeptidase activity. (**A**) HEK293 cells transfected with 3xHA-ACE2-sfGFP were incubated with increasing concentrations of Ang II, Ap-13 or des-Arg^9^-BK for 1 h followed by phenylalanine detection. (**B**) Untransfected or 3xHA-ACE2-sfGFP transfected HEK293 cells were incubated for 1 h with Ang II (25 µM), Ap-13 (25 µM) or des-Arg^9^-BK (75 µM) with or without ACE2 inhibitors DX600 or MLN4760 followed by phenylalanine detection. Unincubated substrates were used for background subtraction. (**C**) Untransfected or 3xHA-ACE2-sfGFP transfected HEK293 cells were incubated with Ang II (25 µM), Ap-13 (25 µM) or des-Arg^9^-BK (75 µM) for various time points up to 4 h followed by phenylalanine detection. (**D**) The free phenylalanine measurements from transfected cells in C were divided by time point-matched results from untransfected cells to determine the magnitude of ACE2 carboxypeptidase activity over background. All assays were performed in triplicate wells and are representative of three independent experiments (n = 9).

In order to assess the catalytic activity of surface ACE2 in HEK cells, transfected cells were presented with a range of concentrations of the three substrates, Ang II, Ap-13, and des-Arg^9^-BK, using production of free phenylalanine as a measure of enzymatic activity (Fig. 2A). Results demonstrate production of free phenylalanine in a dose-dependent manner with a 50% of maximum metabolism (Km) for Ang II and Ap-13 were ~ 22 µM and ~ 25 µM, respectively, similar to the values reported for soluble recombinant ACE2^30^. In contrast, the 50% maximum metabolism of des-Arg^9^-BK was approximately 75 µM, indicating a lower affinity of this substrate. To confirm that the observed substrate metabolism is due to exogenous ACE2, we incubated all three substrates in the presence or absence of two specific ACE2 inhibitors, DX600 or MLN4760. Both inhibitors led to a significant decrease in Phe production (Fig. 2B), implicating metabolism was specific to ACE2.

The time course of exogenous ACE2 carboxypeptidase activity was then tested by incubating all three substrates with both transfected and untransfected HEK cells across various time points up to 4 h. The metabolism of Ang II and Ap-13 reached a plateau after approximately 2 h, whereas the metabolism of des-Arg^9^-BK continued to increase beyond this time point, indicating a slower rate of metabolism (Fig. 2C). Since all substrates undergo limited background metabolism in untransfected cells, we normalized the phenylalanine production from transfected cells by that from untransfected cells for each time point (Fig. 2D). These ratios provide a more specific measure of exogenous ACE2 activity, and demonstrate the highest specific metabolism of Ang II and Ap-13 within 15–30 min of incubation, whereas peak des-Arg^9^-BK metabolism observed at 1 h.

**Figure 3:**
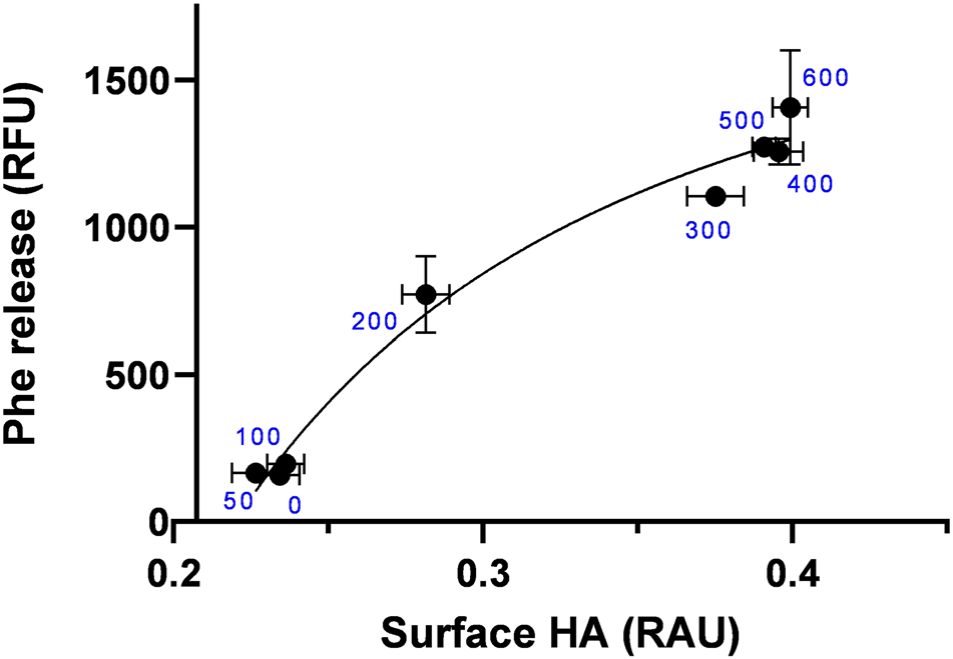
Transfection load impacts on surface receptor expression and carboxypeptidase activity. HEK293 cells were transfected in triplicate with increasing quantities of CMV-3xHA-ACE2-sfGFP plasmid (shown in ng) followed by incubation with 25 µM of Ang II for 30 min and then stained for HA by cell surface ELISA. Assay was performed in triplicate wells and is representative of three independent experiments (n = 9). RAU = relative absorbance units, RFU = relative fluorescence units.

A limitation of transient cell-based assays for assessing exogenous protein function is the variability in number of cells transfected and protein expression levels within each cell due to differential plasmid load. To address these issues, we normalize catalytic activity to total surface expression of ACE2 using antibodies against the N-terminal HA tag. Figure 3 demonstrates the strong correlation between surface ACE2 expression and catalytic activity across a range of surface expression induced by a range of plasmid DNA concentrations used for transfection. This approach enables an accurate measure of the catalytic activity of exogenous surface ACE2 in mammalian cells.

**Figure 4:**
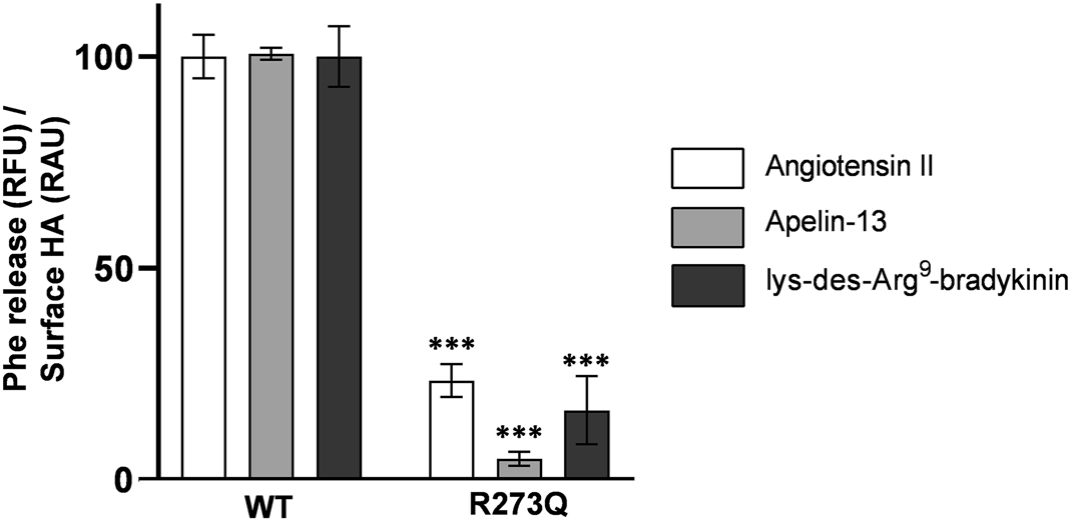
Substrate metabolism per receptor abundance for wildtype and R273Q ACE2. HEK293 cells were transfected in triplicate with WT or R273Q ACE2, incubated with Ang II (25 µM), Ap-13 (25 µM) or des-Arg^9^-BK (75 µM) for 30 min followed by phenylalanine assay and then stained for HA by cell surface ELISA. Phenylalanine values were divided by ELISA values from the same well and then normalized to the WT mean of the same substrate. Assay was performed in triplicate wells and is representative of three independent experiments (n = 9). ***Shows a statistical significance of *P* < 0.001 as determined by a Student’s *t*-test.

Finally, to validate the efficacy of this assay for variant impact on ACE2 catalytic activity, we compared catalytic activity of wildtype ACE2 to a known synthetic catalytically-deficient variant R273Q. HEK cells transfected with ACE2-R273Q exhibited significantly reduced metabolism for all three substrates (Fig. 4).

## Discussion

ACE2 plays a crucial role in various physiological processes, such as regulating blood pressure, inducing vasodilation, promoting antithrombosis and angiogenesis, all of which impact several disease states, including hypertension, cardiovascular, and renal diseases. In addition, ACE2 also has a significant impact on the comorbidities and outcomes associated with SARS-CoV2 infection^4^. Given the high number of ACE2 polymorphisms in the population^24–26^, the high incidence of ACE2-associated disorders^12–23,29^ it is therefore valuable to establish platforms to assess the effects of common genetic variants of ACE2 on its catalytic functions. Although in vitro assays have been developed to study ACE2’s catalytic using recombinant ACE2, these assays are unsuitable for testing large numbers of ACE2 genetic variants due to the associated time and costs of protein purification. Furthermore, recombinant proteins developed in non-mammalian systems typically lack post-translational modifications, such as mammalian cell-specific glycosylation, which can affect protein function. Therefore, developing human cell-based assays is crucial to replicate the physiological features of ACE2 and study the impact of genetic variants^31^.

To develop an assay for exogenous ACE2 catalytic function in physiological conditions that is amenable to high throughput analyses of large numbers of gene variants we have created a vector with both N- and C-terminal tags to distinguish surface from total ACE2 expression in human cell lines. Insertion of an invariant 3xHA tag following the cleaved N-terminal secretion signalling motif of ACE2 enables presentation of the epitope tag on the cell surface. This modification does not appear to affect processing or localization of the protein. Additionally, the C-terminal sfGFP allows rapid monitoring of exogenous ACE2 expression, protein stability and subcellular localization by live-cell flow cytometry and microscopy (not shown) or as shown by confocal microscopy in fixed cells. By measuring and normalizing for ACE2 surface expression this assay measures ACE2 variant catalytic activity independent of variable transfection efficiency or variant impact on protein trafficking or stability. A similar approach to our method may be used to assess endogenous surface ACE2 catalytic activity on a range of cell and tissue types. This would require staining with a ACE2-specific antibody, however would be challenged by the fact that an ACE2 variant may alter interactions with the staining antibody.

Our study design may be affected by a few confounding factors, including the potential contributions of cleaved soluble ACE2 from the surface of transfected HEK cells, serum-born bovine ACE2 and free Phe from the culture medium on catalysis results. To minimize these impacts, multiple PBS washes were performed immediately before substrate addition and the incubation time was kept relatively short (≤ 1 h). There also appears to be low level of background substrate metabolism, particularly for des-Arg^9^-BK, which can be accounted for by subtracting an untransfected control. We have identified optimal transfection and substrate incubation parameters for our assay. The sensitivity and specificity of our assay are comparable to those of Liu and colleagues, who first utilized phenylalanine fluorescence detection to measure Ang II and Ap-13 metabolism^30^. While des-Arg^9^-BK has been known to be metabolized by ACE2^32^, we also demonstrate that this substrate has approximately threefold reduced affinity for ACE2 compared to Ang II and Ap-13.

The convenience of an expression-driven cell-based ACE2 platform permits wide potential applications. We have shown that this tool is amenable for rapid assessment of ACE2 missense variant properties to link genotype to catalytic function, which can be easily scaled up to accommodate large variant libraries. Furthermore, our 3xHA-ACE2-sfGFP cassette could be integrated into a stable cell expression system to screen for pharmaceutical agents impacting ACE2 catalytic functions.

## Methods

### Construction of an ACE2 expression vector

A vector for ACE2 expression with an amino-terminal epitope tag and a carboxy-terminal fluorescent reporter (CMV-ACE2_1-25_-3xHA-3xGGGGS-ACE2_20-805_-3xGGGGS-sfGFP) was generated in house via Gibson assembly. The first 19 amino acids were removed from the full length ACE2 open reading frame (NCBI Reference Sequence: NM_001371415.1) and replaced with ACE2 residues 1–25 plus a 3xHA epitope tag followed by a 3xGGGGS flexible linker sequence (Fig. 1A). The rationale for this design was that since ACE2 normally undergoes N-terminal proteolytic processing after nascent translation and targeting to the plasma membrane, an epitope tag placed immediately after Met1 would be cleaved off. Our design allows the natural leader sequence to be preserved and upon membrane insertion, the 3xHA tag is presented on the surface of membrane-bound ACE2 outside of the cell. Since the protease recognition domain for cleavage of the secretion sequence is unclear, we duplicated amino acids 20–25 both upstream and downstream of the 3xHA tag. The R273Q variant was generated by site-directed mutagenesis using forward and reverse primers 5′-caattttggacaaatctgtactctttgacagttc-3′ and 5′-accccacatatcaccaagcaaatgag-3′ respectively using a Q5 mutagenesis kit (New England Biolabs).

### Cell culture and transfection

HEK293 cells were purchased from the American Type Culture Collection (CRL-1573) and were routinely passaged in low glucose Dulbecco’s Modified Eagle’s Medium supplemented with 10% FBS and 100 U/mL Penicillin–Streptomycin (culture medium). HEK cells were used for a maximum of 15 passages.

### Cell-based assay for ACE2 catalysis

Two days prior to seeding, 24-well cell culture-treated dishes were coated with 80 µL of 250 μg/mL collagen type I from rat tail, 5 μg/mL poly-d-lysine and allowed to fully dry under sterile conditions. Cells were seeded at 9 × 10^5^ per well in 24-well dishes 16–20 h before transfection with 300 ng of ACE2 expression plasmid using X-tremeGENE 9 (Roche) at a ratio of 2 μL to 1 μg DNA. Forty-five hours after transfection, cell culture media were removed and wells were gently washed twice with 350 μL 1× phosphate buffered saline (PBS) containing 10 μM zinc chloride (PBS-Zn). Wash buffer was removed on all wells prior to assay treatments so substrate incubation timing was synchronous. ACE2 substrates and inhibitors dissolved in PBS-Zn were added (150 μL) to wells at their stated concentration and incubated at 37 °C.

### Fluorescent detection of free phenylalanine

Following incubation on cells, substrates were collected at multiple time points and assayed immediately for free Phe using a fluorescence-based detection kit (MilliporeSigma). Substrates collected at varied time points were immediately frozen upon collection and then thawed together so that samples could be measured simultaneously. Through multiple enzymatic steps, Phe is reductively deaminated followed by formation of reduced NADH which reacts with a fluorescent probe. The detection kit was adapted to half volumes scale: 25 μL of cell-incubated substrate was incubated with 1 μL each of enzyme mix and developer solutions plus 23 μL of phenylalanine assay buffer for at 37 °C for 60 min while shielded from light. Fluorescence intensities were measured by single endpoint reads at Ex/Em 535/587 nm using a SpectraMax M2 microplate reader (Molecular Devices).

### Cell-surface enzyme-linked immunosorbent assay (ELISA) for HA

Immediately after substrate removal, cells were fixed with 350 μL 4% formaldehyde dissolved in PBS for 10 min before being quenched with 200 μL PBS saturated with glycine. Fixative was removed and cells were blocked with 150 μL 4% BSA dissolved in PBS (BSA-PBS) for 30 min. Cells were incubated with 120 μL mouse anti-HA (16B12, BioLegend) diluted 1:500 in blocking solution for 60 min before a 10 min wash in PBS-BSA. Cells were incubated with 120 μL sheep anti-mouse secondary antibody conjugated to horse radish peroxidase (GE Healthcare) diluted 1:1000 in PBS-BSA followed by three brief washes with 250 μL PBS. Finally, cells were incubated with 100 μL of 1-Step Ultra-TMB substrate for 30 min according to the manufacturer’s protocol. All steps were carried out at room temperature with gentle rocking. The reaction was halted with 75 μL 2 M sulfuric acid and the combined solution was transferred to a 96 well plate where endpoint absorbance was measured at 450 nm using a SpectraMax M2.

### Confocal microscopy

HEK293 cells were grown on coverslips coated with poly-l-lysine and transfected as described above. One day after transfection, cells were washed once with PBS, blocked in 200 μL PBS containing 2% BSA before incubation overnight at 4 °C with anti-HA antibody (16B12, BioLegend) diluted 1:500 in PBS, 1% BSA. The next morning, cells were washed twice with PBS for 15 min followed by 1 h at room temperature incubation with donkey anti-mouse secondary antibody conjugated to Alexa Fluor 549 (ThermoFisher Scientific) diluted 1:500 in PBS, 1% BSA followed by two additions 15 min washes in PBS. Cells were mounted onto coverslips using Prolong Gold antifade reagent containing 4′,6-diamidino-2-phenylindole (DAPI; ThermoFisher Scientific). Confocal imaging was conducted using a Leica TCS SP5 II Basic VIS system (Leica Microsystems).

### Automated Jess immunoassay

One day after transfection, HEK293 cells were lysed in 1xRIPA buffer (Cell Signaling Technology) containing 1 mM PMSF, 1× protease inhibitor cocktail (ThermoFisher Scientific) and soluble protein fractions were isolated by 10 min centrifugation at 12,000*g* at 4 °C. Using the 12–230 kDa separation module on an automated capillary-based immunoassay system Jess (Protein Simple), approximately 3 µg of lysate from transfected or untransfected HEK cells were analyzed per condition. Samples were incubated with primary antibodies for HA (1:50 dilution, C29F4, Cell Signaling Technology), ACE2 (1:35 dilution, PA520045, ThermoFisher Scientific), ACE2 (1:35 dilution, AF933, R&D Systems), or GFP (1:10 dilution, AF4240, R&D Systems) using the default separation parameters and detected with rabbit or goat chemiluminescent detection modules (Protein Simple).

### Chemicals

All reagents were purchased from MilliporeSigma unless otherwise indicated. ACE2 substrates and inhibitors were dissolved in PBS-Zn and stored at − 20 °C until use.

### Statistical analyses

Data graphing and statistical analyses were performed using Prism 9 (GraphPad). Data in Fig. 4 were analyzed using a Student’s T test as a comparison of means between two groups: WT vs R273Q for each substrate group.

## Data Availability

The datasets used and/or analyzed during the current study are available from the corresponding author upon request.

## References

[1] Hooper NM, Lambert DW, Turner AJ (2020). Discovery and characterization of ACE2—A 20-year journey of surprises from vasopeptidase to COVID-19. Clin. Sci..

[2] Hamming I (2007). The emerging role of ACE2 in physiology and disease. J. Pathol..

[3] Donoghue, M. *et al. A Novel Angiotensin-Converting Enzyme-Related Carboxypeptidase (ACE2) Converts Angiotensin I to Angiotensin *1–9 (2000). 10.1161/01.res.87.5.e1 10969042

[4] Oudit GY, Wang K, Viveiros A, Kellner MJ, Penninger JM (2023). Angiotensin-converting enzyme 2—at the heart of the COVID-19 pandemic. Cell.

[5] Li X (2008). Angiotensin converting enzyme-2 is protective but downregulated in human and experimental lung fibrosis. Am. J. Physiol.-Lung Cell. Mol. Physiol..

[6] Wang R (1999). Angiotensin II induces apoptosis in human and rat alveolar epithelial cells. Am. J. Physiol.-Lung Cell. Mol. Physiol..

[7] Uhal BD, Li X, Xue A, Gao X, Abdul-Hafez A (2011). Regulation of alveolar epithelial cell survival by the ACE-2/angiotensin 1–7/Mas axis. Am. J. Physiol. Lung Cell Mol. Physiol..

[8] Vickers C (2002). Hydrolysis of biological peptides by human angiotensin-converting enzyme-related carboxypeptidase. J. Biol. Chem..

[9] Maguire JJ, Kleinz MJ, Pitkin SL, Davenport AP (2009). [Pyr1]Apelin-13 identified as the predominant apelin isoform in the human heart. Hypertension.

[10] Yamaleyeva LM, Shaltout HA, Varagic J (2016). Apelin-13 in blood pressure regulation and cardiovascular disease. Curr. Opin. Nephrol. Hypertens..

[11] Prado GN (2002). Mechanisms regulating the expression, self-maintenance, and signaling-function of the bradykinin B2 and B1 receptors. J. Cell. Physiol..

[12] Al-Benna S (2020). Association of high level gene expression of ACE2 in adipose tissue with mortality of COVID-19 infection in obese patients. Obes. Med..

[13] Tikellis C (2012). Interaction of diabetes and ACE2 in the pathogenesis of cardiovascular disease in experimental diabetes. Clin. Sci..

[14] Liu C (2018). ACE2 polymorphisms associated with cardiovascular risk in Uygurs with type 2 diabetes mellitus. Cardiovasc. Diabetol..

[15] Narula S (2020). Plasma ACE2 and risk of death or cardiometabolic diseases: A case-cohort analysis. Lancet.

[16] Díez-Freire C (2006). ACE2 gene transfer attenuates hypertension-linked pathophysiological changes in the SHR. Physiol. Genomics.

[17] Crackower MA (2002). Angiotensin-converting enzyme 2 is an essential regulator of heart function. Nature.

[18] Hoffmann M (2020). SARS-CoV-2 Cell entry depends on ACE2 and TMPRSS2 and is blocked by a clinically proven protease inhibitor. Cell.

[19] Verdecchia P, Cavallini C, Spanevello A, Angeli F (2020). The pivotal link between ACE2 deficiency and SARS-CoV-2 infection. Eur. J. Intern. Med..

[20] Khayat AS (2021). ACE2 polymorphisms as potential players in COVID-19 outcome. PLoS ONE.

[21] Brest P, Refae S, Mograbi B, Hofman P, Milano G (2020). Host polymorphisms may impact SARS-CoV-2 infectivity. Trends Genet..

[22] Devaux CA, Rolain J-M, Raoult D (2020). ACE2 receptor polymorphism: Susceptibility to SARS-CoV-2, hypertension, multi-organ failure, and COVID-19 disease outcome. J. Microbiol. Immunol. Infect..

[23] Radzikowska U (2020). Distribution of ACE2, CD147, CD26, and other SARS-CoV-2 associated molecules in tissues and immune cells in health and in asthma, COPD, obesity, hypertension, and COVID-19 risk factors. Allergy.

[24] Cao Y (2020). Comparative genetic analysis of the novel coronavirus (2019-nCoV/SARS-CoV-2) receptor ACE2 in different populations. Cell Discov..

[25] Saengsiwaritt W, Jittikoon J, Chaikledkaew U, Udomsinprasert W (2022). Genetic polymorphisms of ACE1, ACE2, and TMPRSS2 associated with COVID-19 severity: A systematic review with meta-analysis. Rev. Med. Virol..

[26] Darbani B (2020). The expression and polymorphism of entry machinery for covid-19 in human: Juxtaposing population groups, gender, and different tissues. Int. J. Environ. Res. Public Health.

[27] Li J (2022). Polymorphisms and mutations of ACE2 and TMPRSS2 genes are associated with COVID-19: A systematic review. Eur. J. Med. Res..

[28] Huang G (2022). Association of ACE2 gene functional variants with gestational diabetes mellitus risk in a southern Chinese population. Front. Endocrinol..

[29] Horowitz JE (2022). Genome-wide analysis provides genetic evidence that ACE2 influences COVID-19 risk and yields risk scores associated with severe disease. Nat. Genet..

[30] Liu P (2017). A Fluorometric method of measuring carboxypeptidase activities for angiotensin II and apelin-13. Sci. Rep..

[31] Procko E (2020). Deep mutagenesis in the study of COVID-19: A technical overview for the proteomics community. Expert Rev. Proteomics.

[32] Sodhi CP (2018). Attenuation of pulmonary ACE2 activity impairs inactivation of des-Arg 9 bradykinin/BKB1R axis and facilitates LPS-induced neutrophil infiltration. Am. J. Physiol. Lung Cell Mol. Physiol..

